# Supplementary material for: Predicting health behavior in response to the coronavirus disease (COVID-19): Worldwide survey results from early March 2020
Source: PLoS One. 2021 Jan 7;16(1):e0244534. doi: 10.1371/journal.pone.0244534 (PMC7790278; doi:10.1371/journal.pone.0244534)
Supplement: S1 File — (DOCX) [file pone.0244534.s001.docx]

Corona Revised

Start of Block: Corona-related questions

Q111
**The following questions will be about your reaction to the Coronavirus disease.**
 **1. How severe do you think the Coronavirus disease (COVID-19) is?**

- Not severe at all (1)
- Not severe (2)
- A bit severe (3)
- Severe (4)
- Very severe (5)

Q112 **2. How harmful is the Coronavirus disease (COVID-19) to your health?**

- Not harmful at all (1)
- Not harmful (2)
- A bit harmful (3)
- Harmful (4)
- Very harmful (5)

Q113 **3. Do you think that you are susceptible to getting the Coronavirus disease (COVID-19) if you take no preventive measures?**

- Not at all susceptible (1)
- Not really susceptible)
- A bit susceptible (3)
- Quite susceptible (4)
- Very susceptible (5)

Q115 **4. How likely is it that you will be diagnosed in 2020 with one of the following medical conditions?**

|  | Very unlikely (1) | Even (2) | Likely (3) |
| --- | --- | --- | --- |
| Seasonal influenza (1) |  |  |  |
| Diabetes (2) |  |  |  |
| Heart attack (3) |  |  |  |
| Coronavirus disease (COVID-19) (4) |  |  |  |
| HIV or AIDS (5) |  |  |  |

Q117 **5. Are you worried about the Coronavirus disease (COVID-19)?**

- Not at all worried (1)
- Not worried (2)
- A bit worried (3)
- Worried (4)
- Very worried (5)

Q118 **6. Are you scared of the Coronavirus disease (COVID-19)?**

- Not at all scared (1)
- Not scared (2)
- A bit scared (3)
- Scared (4)
- Very scared (5)

Q119 **7. Below are statements, please indicate the degree to which you agree with each of these statements.**

|  | Disagree (1) | Neutral (2) | Agree (3) |
| --- | --- | --- | --- |
| There is nothing we can do about the Coronavirus (1) |  |  |  |
| The threat is exaggerated by the media (2) |  |  |  |
| I will move to a place without the Coronavirus (3) |  |  |  |
| The health authorities in my country should take extra precautionary measures (29) |  |  |  |
| I will stock up and stay indoors (4) |  |  |  |
| It will not be as bad as predicted (5) |  |  |  |
| We will all be completely powerless (6) |  |  |  |
| We just have to accept it (7) |  |  |  |
| The protective steps, taken by my government, are too drastic   (28) |  |  |  |

Q127 **8. Imagine that health authorities’ advice these measures. For each measure, please indicate if you will take this measure.**

|  | definitely  will not take (1) | Probably  will not take (2) | Neutral (3) | Probably  will take (4) | Definitely  will take (5) |
| --- | --- | --- | --- | --- | --- |
| Wash your hands frequently (with an alcohol-based hand rub, or with soap and water). (1) |  |  |  |  |  |
| Maintain at least 1 meter (3 feet) distance between yourself and anyone who is coughing or sneezing (2) |  |  |  |  |  |
| Avoid touching your eyes, nose and mouth (3) |  |  |  |  |  |
| Wear face masks if you are taking care of a person with suspected Coronavirus (4) |  |  |  |  |  |
| Avoid regions/persons with the Coronavirus (5) |  |  |  |  |  |
| Seek medical advice with the onset of symptoms (6) |  |  |  |  |  |

Q120 **9. What have you done so far to prevent yourself from getting the Coronavirus disease (COVID-19)? You can select more than one answer**

- Nothing (1)
- I avoid crowded places (2)
- I maintain distance between myself and people who are coughing and sneezing (3)
- I bought a face mask (4)
- I sought medical consultation (5)
- I avoid regions/persons with the Coronavirus (6)
- I wash my hands frequently (7)
- Other (8)

Q121 **10. If you chose other, please elaborate**

________________________________________________________________

Start of Block: Demographics

Q37 1. Age

- 18-24 (1)
- 25-34 (2)
- 35-44 (3)
- 45-54 (4)
- 55-64 (5)
- 65-74 (6)
- above 75 (7)

Q94 2. Marital Status

- Married (1)
- Widowed (2)
- Divorced (3)
- Separated (4)
- Never married (5)

Q95 3. To which gender identity you most identify?

- Male (1)
- Female (2)
- Transgender Male (3)
- Transgender Female (4)
- Gender variant/Non-conforming (5)
- Not listed (6)
- Prefer not to answer (7)

Q102 4. Education

- Less than high school (1)
- High school graduate (2)
- Some college credit, no degree (3)
- Trade/technical/vocational training (4)
- Bachelor's degree (5)
- Master's degree (6)
- Doctorate degree (7)

Q100 5. Employment

- Employed full time (1)
- Employed part time (2)
- Unemployed looking for work (3)
- Unemployed not looking for work (4)
- Retired (5)
- Student (6)
- Disabled (7)

Q106 6. How would you describe your annual income compared to the average income in your country?

- Much below the average national income (1)
- Below the average national income (2)
- Approximately equal to the average national income (3)
- Above the average national income (4)
- Much above the average national income (5)

Q101 7. Nationality

________________________________________________________________

Q97 8. Ethnic origins

- North American Aboriginal origins (1)
- European origins (2)
- Caribbean origins (3)
- Latin, Central and South American origins (4)
- African origins (5)
- Asian origins (6)
- Oceania origins (7)

Q104 9. City of residence in the past three months

________________________________________________________________

End of Block: Demographics
