# Supplementary material for: Predicting health behavior in response to the coronavirus disease (COVID-19): Worldwide survey results from early March 2020
Source: PLoS One. 2021 Jan 7;16(1):e0244534. doi: 10.1371/journal.pone.0244534 (PMC7790278; doi:10.1371/journal.pone.0244534)
Supplement: Attachment — Submitted filename: Response to Reviewers 1.docx [file pone.0244534.s003.docx]

### November, 13, 2020

Amir H. Pakpour, Ph.D.
Academic Editor
PLOS ONE

Dear Dr. Pakpour,

Thank you and the reviewer for the opportunity to revise and resubmit our manuscript entitled “Predicting health behavior in response to the coronavirus disease (COVID-19): Worldwide survey results from early March 2020” (PONE-D-20-25877). We apologize for the one-day delay in submitting our revision. In the current revision, we attempted to address all the points raised by you and the reviewer in your letter.

In the following, we elaborate on the changes we made in the manuscript in light of your comments (Journal Requirements), followed by the changes we made in response to the reviewer’s comments.

Journal Requirements:

**1. Please ensure that your manuscript meets PLOS ONE's style requirements, including those for file naming. The PLOS ONE style templates can be found at:**

[**https://journals.plos.org/plosone/s/file?id=wjVg/PLOSOne_formatting_sample_main_body.pdf**](https://journals.plos.org/plosone/s/file?id=wjVg/PLOSOne_formatting_sample_main_body.pdf)

[**https://journals.plos.org/plosone/s/file?id=ba62/PLOSOne_formatting_sample_title_authors_affiliations.pdf**](https://journals.plos.org/plosone/s/file?id=ba62/PLOSOne_formatting_sample_title_authors_affiliations.pdf)

We attempted to style our manuscript so it will meet PLOS ONE style requirements.

**2. In the ethics statement in the Methods and online submission information, please specify the type of informed consent that was obtained from the participants (for instance, written or verbal, and if verbal, how it was documented and witnessed).**

Before starting the questionnaire participants read in the Qualtrics software a written consent form and approved it by selecting the “Yes” button. This is now clarified on p. 4 (lines 83-85).

**3. Please provide further details on sample size and power calculations.**

Two major analyses were conducted in the study. The first analysis was a multivariate logistic regression analysis. Details on the power calculation for the regression are provided on p. 7 (lines 159-161): “An a-priori power analysis (G*Power, [10]) for linear multiple regression, fixed model, R^2^ increase with an alpha of 0.05, power of 0.80, and 20 predictors revealed that the sample size is sufficient to detect a small-size effect (*f^2^*=.02).” The second analysis was a partial least squares structural equation modeling (PLS-SEM). Details on the PLS-SEM are also provided (p. 8, lines 167-168):” Power calculation determined that the sample size is large enough to detect a small-size effect (0.80 power, alpha of .05 and 6 predictors, see [13]).” Finally, to address the concern of Type-I error inflation in the chi-square, t-tests and ANOVAS we restricted our conclusions to a significance level of less than .0005 (see p.7, lines 146-147).

**4. Please include additional information regarding the survey or questionnaire used in the study and ensure that you have provided sufficient details that others could replicate the analyses. For instance, if you developed a questionnaire as part of this study and it is not under a copyright more restrictive than CC-BY, please include a copy, in both the original language and English, as Supporting Information.**

The survey was added as Supporting Information.

**5. In the Methods, please discuss whether and how the questionnaire was validated and/or pre-tested. If this did not occur, please provide the rationale for not doing so.**

We used the format of previous surveys that investigated similar pandemics in the past. Also, we referred to the CDC website to insert items specific to the current pandemic. We pre-tested the study using a small sample of 30 participants. After perusing the data and not finding any inconsistencies, misunderstandings, or other problems, we included these participants in the total sample. To clarify this point, see p. 5, lines 95-99:” The survey questions were based on studies that assessed risk perception and precautionary behaviors of the general public in previous pandemic outbreaks (e.g., SARS, avian influenza [9]). Items specific to COVID-19, such as preventive measures, were taken from the website of the Centers for Disease Control and Prevention ([www.cdc.gov](http://www.cdc.gov)).”

**6. Please include captions for your Supporting Information files at the end of your manuscript, and update any in-text citations to match accordingly. Please see our Supporting Information guidelines for more information**: http://journals.plos.org/plosone/s/supporting-information.

Done.

Responses to Reviewer 1

**1. There are some papers using behavioral model to examine the preventive behaviors during the COVID-19 outbreak period, the authors should cite these papers and have comparisons with these papers as they are relevant to this topic. Please see the following.
Chang, K.-C., Strong, C., Pakpour, A. H., Griffiths, M. D., & Lin, C.-Y. (2020). Factors related to preventive COVID-19 infection behaviors among people with mental illness. Journal of the Formosan Medical Association.** [**https://doi.org/10.1016/j.jfma.2020.07.032**](https://doi.org/10.1016/j.jfma.2020.07.032) **Lin, C.-Y., Imani, V., Majd, N. R., Ghasemi, Z., Griffiths, M. D., Hamilton, K., Hagger, M. S., & Pakpour, A. H. (2020). Using an Integrated Social Cognition Model to Predict COVID-19 Preventive Behaviors. British Journal of Health Psychology.** [**https://doi.org/10.1111/bjhp.12465**](https://doi.org/10.1111/bjhp.12465) **Lin, C.-Y., Broström, A., Griffiths, M. D., & Pakpour, A. H. (2020). Investigating mediated effects of fear of COVID-19 and COVID-19 misunderstanding in the association between problematic social media use and distress/insomnia. Internet Interventions, 21, 100345. doi:10.1016/j.invent.2020.100345
Hagger, M. S., Smith, S. R., Keech, J. J., Moyers, S. A., & Hamilton, K. (2020). Predicting Social Distancing Intention and Behavior During the COVID-19 Pandemic: An Integrated Social Cognition Model. Annals of Behavioral Medicine.**

[**https://doi.org/10.1093/abm/kaaa073**](https://doi.org/10.1093/abm/kaaa073)

We referred to these very relevant references in several locations the Discussion in order to compare their results with ours. See, pp. 19-20 (lines 376-388) and p. 21 (lines 413-420).

**2. The authors should use the following references to portray the importance and conditions of preventive behaviors during COVID-19 outbreak. Specifically, Rieger in his two papers describes the intention to perform some preventive behaviors among Germany. Shrivastava and Shrivastava mentioned the shortage of personal protective equipment during the COVID-19 outbreak period. Therefore, with proper implementation of preventive behaviors, the problem of shortage in personal protective equipment may be resolved (i.e., the population may not be panic on the outbreak and not crazily purchase the personal protective equipment). Lin and Cheng discussed the effectiveness of successful government policy. Therefore, the findings presented in the current study may take reference from Lin and Cheng to activate governments in doing some reactions timely and immediately.
Rieger MO. Triggering altruism increases the willingness to get vaccinated against COVID-19. Soc Health Behav 2020;3:78-82
Rieger MO. To wear or not to wear? Factors influencing wearing face masks in Germany during the COVID-19 pandemic. Soc Health Behav 2020;3:50-4
Shrivastava SR, Shrivastava PS. COVID-19 pandemic: Responding to the challenge of global shortage of personal protective equipment. Soc Health Behav 2020;3:70-1
Lin MW, Cheng Y. Policy actions to alleviate psychosocial impacts of COVID-19 pandemic: Experiences from Taiwan. Soc Health Behav 2020;3:72-3**

We implemented the reviewer’s suggestion and the references provided in the following paragraph in the Discussion (p. 18, lines 353-357): “Effects of demographic variables on the different constructs of health behavior examined in the study are ostensible. The relationship of various demographic variables to different precautionary measures emphasizes that the authorities' health behavior campaigns should not be uniform. As recent findings indicate, healthcare providers should implement tailor-made policies to maximize the efficacy of COVID-19 preventive measures, and its maximal propagation to all quarters of the population [17-19]”.

**3. I wondering why the authors did not use any assessments that have been designed for COVID-19. Specifically, Ahorsu et al. have developed the Fear of COVID-19 Scale; Lee has developed the Coronavirus Anxiety Scale and Obsession with COVID-19 Scale; Taylor et al. have developed the COVID Stress Scales; Chang et al. have developed Preventive COVID-19 Infection Behaviors Scale. The authors should at least acknowledge this as one of the limitations. That is, mentioning in their Limitation section that these are the available instruments to assess COVID-19 impacts with proper citations.**

The survey was conducted in the early days of the COVID-19 pandemic (between March 2-14). The assessments that the reviewer mentions were published only later: Ahorsu et al, 2020 was published online on March 27, 2020, Lee (2020) coronavirus anxiety scale was published online on April 16, 2020, Lee (2020) Obsession with COVID-19 Scale was posted online on April 27, Taylor et al., COVIS Stress scale was available online on May 4, 2020, Chang et al., (2020) Preventive COVID-19 Infection Behaviors Scale (PCIBS) was accepted for publication on July 24, 2020. In the Discussion, we acknowledge the existence of these scales and recommend their use in future studies (p. 21, lines 417-420): “Also, since the present survey was launched, several scales were developed that assess both COVID-19 related health behavior [25] and specific predictors such as fear [30], anxiety [31], obsession [32], and stress [33]. Their use in future studies addressing the issue of health behavior is warranted.”.

**4. As the sample is consisted of different ethnicity groups, I wonder whether the authors have all different languages used in the present study. Or, the authors only use English for all the measures? This information should be clearly indicated and if the latter is the truth, this should be acknowledged as one of the limitations.**

The survey was written in English. We acknowledge this limitation in the Discussion (p. 20, lines 402-403):” Moreover, we administered the survey in English. Although we used plain language, it may have proved some challenge to non-native English speakers.”

**5. In Figure 1, it is unclear what “Demographic 1” and “Demographic 2” indicate.**

Since Figure 1 is a theoretical model we did not know in advance which demographic variables will be included in the model. Therefore, we wrote Demographic 1 and 2 to denote the existence of demographic variables in the model. In order to clarify the model, we changed the terms Demographic 1 and 2 to the term Demographic variables.

**6. Please report all the p-values instead of using symbols to indicate whether the p-value is over 0.05, smaller 0.05, smaller 0.01, or smaller 0.001. For those p-values smaller than 0.001, the authors can use “<0.001” to present.**

P-values are now reported throughout the Figures.

**7. In Table 4, please provide the SE of the unstandardized coefficient. Also, please provide the R square and adjusted R square for each step.**

First, we corrected a typo and renamed Table 4 as Table 5. We also added all the information requested by the reviewer to Table 5.

**8. I am not sure whether the authors have adjusted their type 1 error in all the t-tests and ANOVAs. It seems that they did a lot of inferential testing and the chance of type 1 error inflation is high.**

We have taken into account the risk of type 1 inflation error due to the high number of statistical tests conducted. We therefore considered as significant results whose alpha was smaller than .0005. This is mentioned now on p. 7, lines 146-147: “To avoid false positives resulting from multiple comparisons the alpha Type-I error was set >0.0005”.

## We would like to thank you and the reviewer for the many valuable comments that improved our manuscript. We hope that this revised version will be suitable for publication in *PLOS ONE*.

## Sincerely,

## David Anaki & Jamie Singer
